# A chimeric vaccine derived from Australian genotype IV Japanese encephalitis virus protects mice from lethal challenge

**DOI:** 10.1038/s41541-024-00903-2

**Published:** 2024-07-31

**Authors:** Jessica J. Harrison, Wilson Nguyen, Mahali S. Morgan, Bing Tang, Gervais Habarugira, Henry de Malmanche, Morgan E. Freney, Naphak Modhiran, Daniel Watterson, Abigail L. Cox, Kexin Yan, Nicholas K. Y. Yuen, Dylan H. Bowman, Peter D. Kirkland, Helle Bielefeldt-Ohmann, Andreas Suhrbier, Roy A. Hall, Daniel J. Rawle, Jody Hobson-Peters

**Affiliations:** 1 School of Chemistry and Molecular Biosciences, The University of Queensland, St Lucia, Brisbane, 4072 Australia; 2 QIMR Berghofer Medical Research Institute, Brisbane, 4029 Australia; 3 Australian Infectious Disease Research Centre, Brisbane, 4072, 4029 Australia; 4 School of Veterinary Science, The University of Queensland, Gatton, QLD 4343 Australia; 5 Department of Primary Industries, Elizabeth Macarthur Agricultural Institute, Menangle, NSW 2568 Australia

**Keywords:** Recombinant vaccine, Live attenuated vaccines

## Abstract

In 2022, a genotype IV (GIV) strain of Japanese encephalitis virus (JEV) caused an unprecedented and widespread outbreak of disease in pigs and humans in Australia. As no veterinary vaccines against JEV are approved in Australia and all current approved human and veterinary vaccines are derived from genotype (G) III JEV strains, we used the recently described insect-specific Binjari virus (BinJV) chimeric flavivirus vaccine technology to produce a JEV GIV vaccine candidate. Herein we describe the production of a chimeric virus displaying the structural prM and E proteins of a JEV GIV isolate obtained from a stillborn piglet (JEV_NSW/22_) in the genomic backbone of BinJV (BinJ/JEV_NSW/22-_prME). BinJ/JEV_NSW/22-_prME was shown to be antigenically indistinguishable from the JEV_NSW/22_ parental virus by K_D_ analysis and a panel of JEV-reactive monoclonal antibodies in ELISA. BinJ/JEV_NSW/22-_prME replicated efficiently in C6/36 cells, reaching titres of >10^7^ infectious units/mL - an important attribute for vaccine manufacture. As expected, BinJ/JEV_NSW/22-_prME failed to replicate in a variety of vertebrate cells lines. When used to immunise mice, the vaccine induced a potent virus neutralising response against JEV_NSW/22_ and to GII and GIII JEV strains. The BinJ/JEV_NSW/22-_prME vaccine provided complete protection against lethal challenge with JEV_NSW/22_, whilst also providing partial protection against viraemia and disease for the related Murray Valley encephalitis virus. Our results demonstrate that BinJ/JEV_NSW/22-_prME is a promising vaccine candidate against JEV.

## Introduction

Japanese encephalitis virus (JEV, *Orthoflavivirus japonicum*) is a mosquito-borne flavivirus and the leading cause of vaccine-preventable viral encephalitis in Asia and the western Pacific^1^. JEV is transmitted by mosquitoes, primarily of the *Culex* species and is sustained in an enzootic cycle with wading birds and pigs as amplifying hosts^2^. There are five phylogenetically distinct genotypes (GI-GV) of JEV, each with its own geographic distribution pattern^3^.

JEV has demonstrated a strong propensity to spread and establish in new areas^2^. Within Australia, JEV (GII) was first detected in 1995 when three cases of encephalitis were reported on the outer islands of the Torres Strait, resulting in two deaths^4,5^. Further incursions of JEV GII occurred in 1998^6^, while several GI isolates were identified in mosquitoes captured in the Torres Strait and the Northern Peninsula Area of Cape York, north-eastern Australian mainland in 2000^7,8^ and 2004^9^, respectively. Despite this, the virus never became established on the Australian mainland. However, a fatal case caused by JEV GIV in the Tiwi Islands in 2021, saw the expansion of this genotype for the first time outside of Indonesia^10^, with the exception of a GIV strain (strain VN113) isolated from a human in Vietnam in 1979 (Genbank Accession Number: KU705228.1). Subsequently, a genotype IV strain of JEV (JEV_NSW/22_) caused an unprecedented and widespread outbreak of disease in pigs and humans in Australia^11^. The virus was detected in infected pigs and mosquitoes in most mainland states of Australia, extending as far south as Victoria and involving >80 piggeries^12^. To date, 45 human cases have been reported, resulting in 7 deaths^13^.

Two JEV vaccines are commercially available in Australia for human use, including an inactivated vaccine (Ixiaro/JEspect) based on an attenuated strain of JEV (SA-14-14-2) and a chimeric live attenuated vaccine (IMOJEV) based on the genome backbone of the vaccine strain of YFV (17D) and incorporating the prM & E genes from JEV SA-14-14-2^14,15^. A live attenuated vaccine based on SA-14-14-2 is licensed in China and some other Asian countries^16^. However, an urgent call for prevention measures during the Australian outbreak highlighted a limited supply of the commercially available vaccines for use in humans and the absence of a vaccine approved for veterinary use in this country^11^.

While JEV infection in adult pigs causes no overt clinical signs, it can cause reproductive issues, with infection of pregnant sows often resulting in death of the fetus or severe abnormalities in newborn piglets, whilst infection of boars can result in prolonged infertility^17^. In this context, the Australian pork industry was substantially affected in 2022, with JEV infection resulting in fertility and production losses on pig farms^18^. Thus, a vaccine for veterinary use is pertinent for the protection of farmed pigs and disrupting their involvement in the enzootic cycle, as well as for the protection of horses, which, although they are dead-end hosts, can develop clinical manifestations resembling those seen in humans^19,20^.

The Australian mosquito season of 2022/2023 also saw a surge in Murray Valley encephalitis virus (MVEV) cases^21^. This was the first outbreak of MVEV involving humans in south-eastern Australia (Victoria) since 1974^22^, with 26 cases and reports of 7 deaths Australian-wide during this season (Victorian Health. https://www.health.vic.gov.au/infectious-diseases/mosquito-borne-diseases (2023); Government of Western Australia. https://www.health.wa.gov.au/Media-releases/2023/March/WA-child-from-West-Kimberley-dies-of-Murray-Valley-Encephalitis; https://www.theguardian.com/australia-news/2023/feb/14/health-alert-as-woman-dies-of-murray-valley-encephalitis-in-northern-territory; Australian Government Department of Health and Aged Care, https://www.health.gov.au/our-work/nndss (2024)). Endemic only to Australia, Papua New Guinea and Papua, MVEV is similarly transmitted predominantly by *Culex* spp. mosquitoes and maintained in an enzootic transmission cycle involving waterbirds^23^. Humans and horses are at risk of developing clinical signs upon infection with MVEV often mirroring the presentation of JEV, including fever, headache, seizures and confusion^24,25^. As no human or veterinary vaccines against MVEV are available, evidence for cross-protection afforded by JEV vaccination is important in the Australian context given the potential for co-circulation^22^, in addition to confirmation that prior JEV vaccination does not cause the phenomenon of antibody-dependent enhancement^26^.

To address these issues, we used a patented recombinant vaccine platform based on the insect-specific flavivirus Binjari virus (BinJV)^27^, which we have previously shown to have a robust safety profile and accurately mimic the virion antigenic structure of a range of pathogenic flaviviruses^27–32^. We constructed a chimeric virus that authentically displayed the immunogenic structural proteins (prM and E) of the JEV_NSW/22_ outbreak strain. This chimeric virus (BinJ/JEV_NSW/22-_prME) was shown to be a safe and effective vaccine against homotypic JEV challenge in a mouse model and provided partial cross-protection against MVEV.

## Results

### Generation and characterisation of BinJ/JEV_NSW/22-_prME

**Fig. 1:**
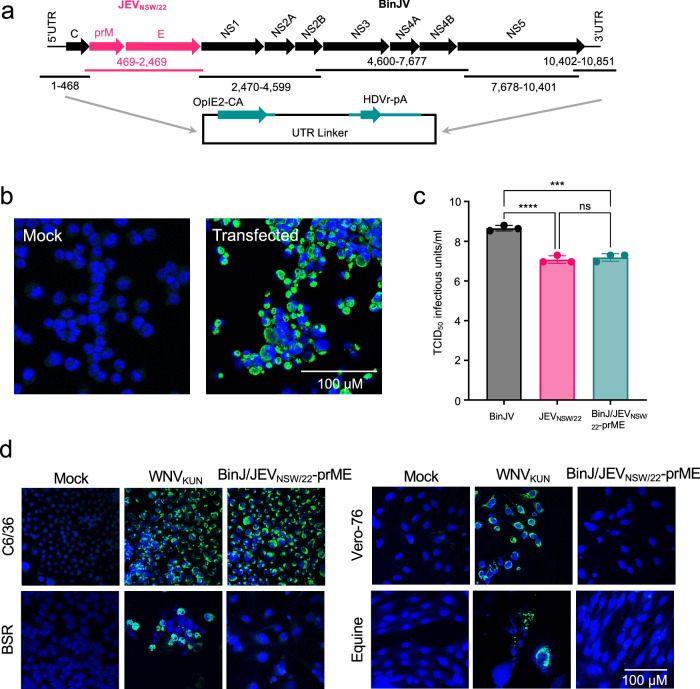
Generation and characterisation of the BinJ/JEV_NSW/22_-prME chimera. **a** Schematic of the circular polymerase extension reaction (CPER) strategy to generate infectious DNA of chimeric BinJ/JEV_NSW/22_-prME. The prME genes of JEV_NSW/22_ (pink arrows) were inserted into the BinJV backbone (black arrows) (replacing the BinJV prME). OpIE2-CA, a modified Orgyia pseudotsugata multicapsid nucleopolyhedrosis virus immediate-early 2 promoter; HDVr-pA, hepatitis delta virus ribozyme–poly A, with the ribozyme autocleavage providing an authentic 3′ untranslated region (UTR). **b** IFA analysis of mock (left) and BinJ/JEV_NSW/22_-prME CPER (right) transfected C6/36 cells fixed 7 days post-transfection and immunolabeled with anti-flavivirus NS1 mAb 4G4 to confirm recovery of replicating virus (green signal). **c** Growth of chimera was analysed by infecting C6/36 monolayers in triplicate with BinJV, JEV_NSW/22_ or BinJ/JEV_NSW/22_-prME at an MOI of 0.1, before titrating on C6/36 monolayers in triplicate and determining viral titres by TCID_50_. Statistics were performed by one-way ANOVA whereby *p* = <0.0001 (****) or <0.005 (***). **d** IFA analysis by confocal microscopy of mock, West Nile virus, Kunjin subtype (WNV_KUN_, a mammalian cell infection control) and BinJ/JEV_NSW/22_-prME virus-infected C6/36 cells and mammalian cells at an MOI of 1. Cells were fixed and immunolabelled 5 days after infection. Mammalian cells: BSR (baby hamster kidney), Vero-76 cells (African green monkey kidney) and primary equine dermal fibroblasts. Virus replication was detected with anti-NS1 mAb 4G4 (green signal) and cell nuclei were stained with Hoechst 33342 (blue). Images taken at x40 magnification. Scale bar in (**b**) and (**d**) represents 100 μM.

A chimeric virus displaying the prM and E proteins of the Australian New South Wales 2022 (NSW/22) GIV outbreak strain in the genomic backbone of the insect-specific flavivirus, BinJV, was created using Circular Polymerase Extension Reaction (CPER) as previously described (Fig. 1a)^27^. A viable virus was recovered from transfected C6/36 cells and designated BinJ/JEV_NSW/22-_prME (Fig. 1b). The identity of the recovered chimeric virus was confirmed by deep sequencing. In addition to an intended silent mutation introduced at position 480 of the genome (located in prM), incorporated via a primer during amplification of the JEV prM/E insert, three other nucleotides differed from what was anticipated, but did not result in amino acid changes (Supplementary Table 1). Replication kinetics demonstrated that the recovered chimera grew to a high titre (10^7.2^ TCID_50_/mL), similar to that achieved by the wild-type JEV_NSW/22_, (10^7.08^ TCID_50_/mL) in C6/36 mosquito cells (Fig. 1c).

### BinJ/JEV_NSW/22-_prME does not replicate in vertebrate cells

Previously, we have shown that chimeric viruses derived from BinJV do not replicate in vertebrate cells, consistent with an insect-specific phenotype^27^. To ensure that BinJ/JEV_NSW/22-_prME retained the insect host-restricted phenotype, monkey (Vero 76) and rodent (BSR) cell lines known to be highly permissive to flavivirus replication and partially deficient in the interferon response^33,34^ were assessed for BinJ/JEV_NSW/22_-prME infection and replication. A primary equine cell line was also assessed due to the capacity of JEV to cause disease in equines^35^. The cell monolayers were inoculated with BinJ/JEV_NSW/22_-prME and incubated for 7 days before assessment for viral replication by IFA using the pan-flavivirus NS1 antibody, 4G4^36^. No viral replication was detected in the inoculated vertebrate cell lines, while C6/36 cells showed high levels of NS1 expression, indicative of viral replication (Fig. 1d). These data supported the conclusion that productive replication of BinJ/JEV_NSW/22-_prME is restricted to mosquito cells.

### BinJ/JEV_NSW/22-_prME particles show typical flavivirus morphology

**Fig. 2:**
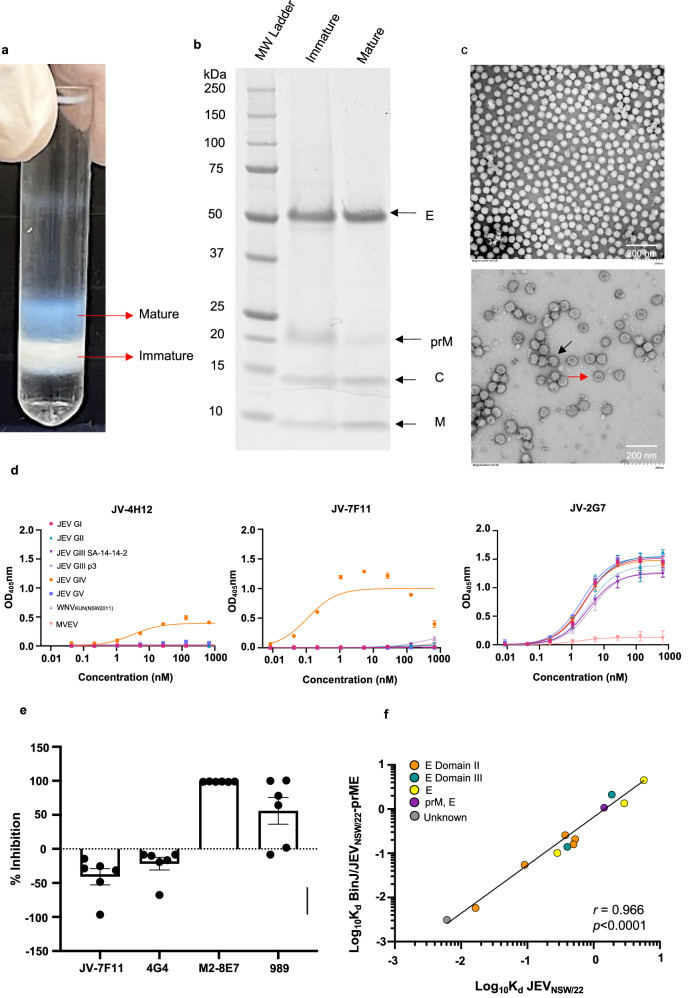
BinJ/JEV_NSW22_-prME vaccine antigen purification. **a** BinJ/JEV_NSW/22_-prME was purified via a potassium tartrate gradient, sedimenting as a wide opalescent blue band (mature virions) or white band (immature virions). **b** SDS-PAGE (4–12%) analysis of gradient purified BinJ/JEV_NSW/22_-prME. Flavivirus structural proteins (pre-membrane (prM), capsid (C), membrane (M) and envelope (E)) are indicated. **c** Negative-stain TEM image of mature (upper panel, x20, 000 magnification) and immature (lower panel, x25, 000 magnification) BinJ/JEV_NSW/22_-prME virions. Scale bar represents 200 nm. For lower panel, example of mature virion (red arrow) and immature virion (black arrow) indicated. **d** ELISA curves for indicated purified anti-JEV mAbs using C6/36 fixed cells that had been infected with BinJV-based JEV chimeras GI-V, wild-type WNV_KUN_ and MVEV or mock-infected as the antigen. **e** Competitive ELISA analysis using saturating concentrations of mAbs listed on the X axis and detection of binding of mAb JV-4H12. **f** Kd values for binding of mAbs to BinJ/JEV_NSW/22_-prME and the corresponding JEV_NSW/22_; each dot represents one mAb (mAbs are described in Table S4). Yellow, E-specific mAbs; orange, E domain II–specific mAbs; green, E domain III–specific mAbs; purple, prM and E-specific mAbs, grey, unspecified prM/E-reactive mAbs. Statistics were performed using Pearson correlations.

Purification of BinJ/JEV_NSW/22-_prME via a potassium tartrate gradient yielded distinct opalescent blue (upper band) and white (lower band) fractions (Fig. 2a). When fractions corresponding to each band were analysed by SDS-polyacrylamide gel electrophoresis (SDS-PAGE), and Sypro Ruby protein staining, bands corresponding to the correct size (kDa) of prM and E structural proteins were clearly visible from the white band fraction, indicative of immature virions, whereas only M and E were clearly evident in the blue fraction, indicative of mature virions (Fig. 2b). Assessment of the intensity of the E bands against a BSA standard indicated a yield of 2 mg of E protein derived from mature virions per L of viral supernatant was obtained (Supplementary Fig. 1). Negative staining transmission electron microscopy of each fraction revealed predominantly smooth (mature) (Fig. 2c, upper) or spiky (immature) (Fig. 2c, lower) spherical virions of ~50 nm in diameter, typical of flavivirus virion morphology. The purified material from the opalescent blue band (mature particles) was used for subsequent immunisation and challenge studies, consistent with our previous studies^29,37^.

### BinJ/JEV_NSW/22-_prME particles are antigenically similar to wild-type JEV_NSW/22_

Flavivirus virus-like particles (VLPs) produced recombinantly by baculovirus or mammalian expressions systems can display structural irregularities, resulting in quaternary epitopes, in particular, not being authentically displayed^38,39^. The antigenic authenticity of BinJ/JEV_NSW/22-_prME was confirmed using a panel of mAbs (Supplementary Table 2) that are reactive to the prM and/or E proteins (multiple domains) of JEV. To bolster the available mAbs to assess the epitope presentation of the chimera, new mAbs were produced through immunisation of mice with BinJ/JEV_NSW/22-_prME. Of the new panel, 2 mAbs (JV-4H12, JV-7F11) specifically bound JEV GIV (Fig. 2d, Supplementary Table 2), with no cross-reactivity to other JEV genotypes detected and through competitive ELISA, confirmed to bind discrete epitopes, due to the inability of mAb JH-7F11 to inhibit the binding of JV-4H12 (Fig. 2e). Interestingly, mAb JV-4H12 binding was completely inhibited by E protein domain III (EDIII) binder, mAb M2-8E7^40,41^ (100% inhibition, Fig. 2e), indicating shared contact residues. Moderate inhibition of mAb JV-4H12 binding was also shown with mAb 989^42^, which by Western blot, was shown to bind both prM and E proteins of JEV (Supplementary Fig. 2). To confirm the presence of adequate antigen input in the dissociation constant (K_d_) ELISA system, mAb JV-2G7 bound all JEV strains similarly. The specific binding of mAbs JV-4H12 and JV-7F11 to JEV GIV underscore the antigenic distinction of JEV GIV from the other genotypes.

The apparent dissociation constants (K_d_) of the panel of mAbs for the parental JEV_NSW/22_ and the BinJ/JEV_NSW/22-_prME chimera were determined (Fig. 2f, Supplementary Fig. 3, Supplementary Tables 3 and 4). Pearson correlations indicated that the apparent K_d_ values for the mAb binding between the two viruses were nearly identical (Fig. 2f). These data indicated a high level of antigenic authenticity for BinJ/JEV_NSW/22-_prME chimeric particles.

### Immunisation with BinJ/JEV_NSW/22-_prME protects *Ifnar*^*-/-*^ mice from lethal challenge with wild-type JEV_NSW/22_

**Fig. 3:**
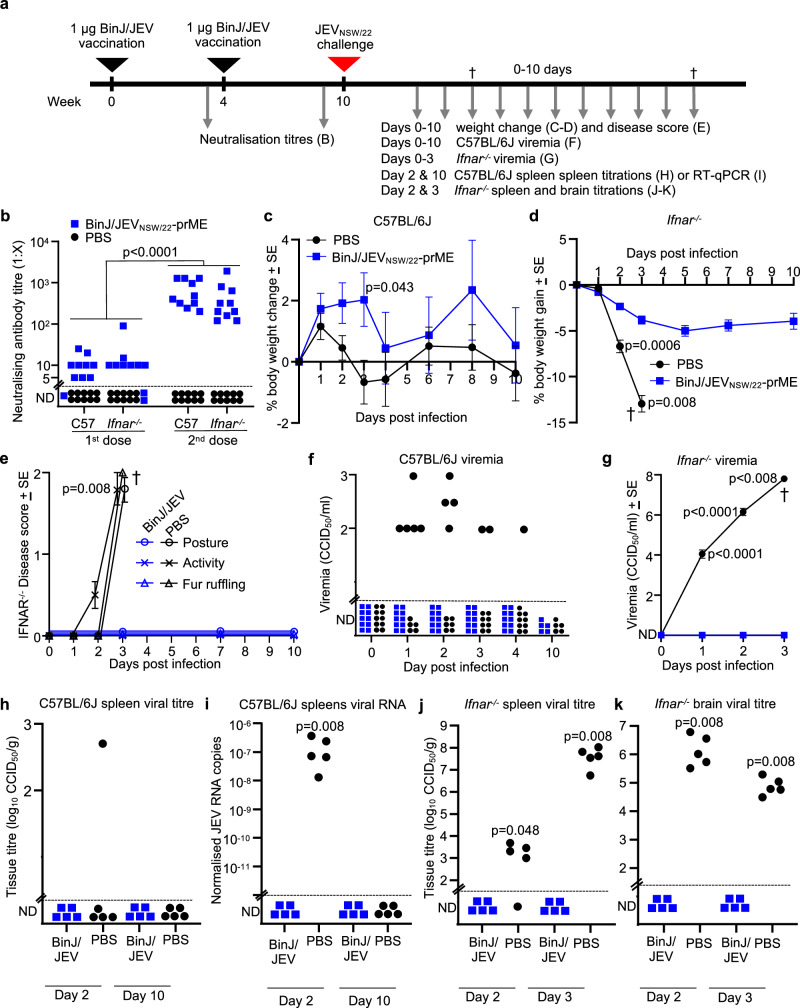
BinJ/JEV_NSW/22-_prME vaccination and challenge. **a** Timeline of BinJ/JEV_NSW/22_-prME vaccinations, JEV_NSW/22_ challenge, and sample/data collections. Silver arrows indicate sample/data collection timepoints. **b** Serum end-point neutralising antibody titres after one (1st dose) and two (2nd dose) vaccinations. Limit of quantification 1 in 5. (Statistics by Kolmogorov-Smirnov tests). **c** Mean percent C57BL/6J mouse body weight change relative to day 0 for each mouse (*n* = 10 per group 0-2 dpi, *n* = 5 per group 3–10 dpi). Differences only reached significance at 3 dpi, statistics by *t*-test (significance was not reached on any other day, or ranges of days by repeated measure ANOVA tests). ND not detected. **d** As for C for *Ifnar*^*-/-*^ mice. Statistics by Kolmogorov-Smirnov exact tests (2 and 3 dpi). **e** Clinical disease scores for posture (circles), activity (crosses) and fur ruffling (triangles) were monitored in *Ifnar*^*-/-*^ mice. *N* as in (**c**). † Ethically defined end point reached for euthanasia. Statistics by Kolmogorov-Smirnov exact tests (*n* = 5 per group), *p* = 0.013 for each disease parameter. **f** Post-challenge viremia in C57BL/6J mice determined by TCID_50_ assays. Viremia for individual mice at each time point. Limit of detection was 2 log_10_TCID_50_/ml. ND not detected. In the PBS group no viremia was detected on any day in 3/10 mice. Statistics by Kolmogorov-Smirnov exact test for 1–4 dpi (*n* = 30 serum samples per group; 30 vs. 18 ND). **G** Post-challenge viremia in *Ifnar*^*-/-*^ mice (*n* = 10 per group 0-2 dpi, *n* = 5 per group 3 dpi). **h** Tissue titres for C57BL/6J spleens. Limit of detection ≈2.7 log_10_TCID_50_/g. **i** RT-qPCR of JEV RNA in C57BL/6J spleens. JEV RNA copies are normalized to *Rpl13a*. Limit of detection ≈10^-12^ JEV/*Rpl13a* copies. **j** Spleen and (**k**) brain tissue titres in *Ifnar*^*-/-*^ mice 2 and 3 dpi.

C57BL/6J and *Ifnar*^*-/-*^ mouse models of JEV infection^43^ were used to evaluate BinJ/JEV_NSW/22-_prME chimeric viral particles as a vaccine antigen. In the C57BL/6J model, JEV_NSW/22_ produces a measurable viremia in 6 week old mice, however infection is non-lethal, with lethal neurotropism significantly rarer than for JEV GII and GIII^43^. In *Ifnar*^*-/-*^, JEV_NSW/22_ produces a robust viremia and infection is lethal^43^. Female C57BL/6J or *Ifnar*^*-/-*^ mice received two i.m. vaccinations ~4 weeks apart with 1 μg of unadjuvanted BinJ/JEV_NSW/22-_prME in 50 μL of PBS, or were injected with PBS only (Fig. 3a). Serum end point neutralizing antibody titers were determined before the second vaccination and before JEV_NSW/22_ challenge (Fig. 3a). Most BinJ/JEV_NSW/22-_prME vaccinated mice had low levels of serum neutralising antibodies against JEV_NSW/22_ after one vaccination, but this increased significantly by ≈2 logs after the second vaccination (Fig. 3b, *p* < 0.0001). There was no significant difference in vaccine-induced neutralising antibody titres between C57BL/6J and *Ifnar*^*-/-*^ mice. Any inherent self-adjuvanting activity for the BinJV chimeras^27,30,32,44^ would thus not appear to be overly dependent on the type I IFN receptor, although IFNα/β-independent interferon-stimulated gene (ISG) induction may still be involved^45,46^.

Post-challenge changes in body weight were minimal in C57BL/6J mice, although on 3 dpi sham immunised (PBS-injected) and infected mice showed a marginal (≈3%), but significantly greater, body weight loss than BinJ/JEV_NSW/22_-prME vaccinated mice (Fig. 3c, *p* = 0.043). In contrast PBS-injected *Ifnar*^*-/-*^ mice showed rapid weight loss (>10%), whereas BinJ/JEV_NSW/22-_prME vaccinated *Ifnar*^*-/-*^ mice were protected against such weight loss, with significant protection seen on 2 and 3 dpi (Fig. 3d, *p* = 0.0006 day 2, *p* = 0.008 day 3). BinJ/JEV_NSW/22-_prME vaccinated mice were also completely protected against overt clinical disease manifestations, with PBS-vaccinated mice showing abnormal posture (hunching), activity loss and fur ruffling reaching ethically defined end points on 3 dpi and required euthanasia (Fig. 3e).

BinJ/JEV_NSW/22-_prME vaccination provided complete protection against detectable viremia in C57BL/6J mice; vaccinated animals showed no viremia on any day whereas in PBS-injected mice, 7/10 animals had a detectable viremia on at least 1 day, with no detectable viremia in 3/10 mice on any day (Fig. 3f). BinJ/JEV_NSW/22-_prME vaccination also provided complete and significant protection against viremia in *Ifnar*^*-/-*^ mice (Fig. 3g, *p* < 0.0001 days 1–2, *p* < 0.008 day 3). Although infectious virus in spleens of C57BL/6J mice was usually undetectable (Fig. 3h), RT-qPCR revealed complete and significant BinJ/JEV_NSW/22-_prME-mediated protection against detectable viral RNA in spleen at 2 dpi (Fig. 3i, *p* = 0.008). BinJ/JEV_NSW/22-_prME vaccination also provided complete protection against virus replication in spleens of *Ifnar*^*-/-*^ mice at 2–3 dpi (Fig. 3j). Brains were similarly protected at 2–3 dpi (Fig. 3k), although virus detection in brains of *Ifnar*^*-/-*^ mice likely represents virus in the blood vessels rather than productive brain infection^43^. Taken together, these data illustrate that BinJ/JEV_NSW/22-_prME vaccination provides robust protection against infection and disease after JEV_NSW/22_ challenge in both C57BL/6J and *Ifnar*^*-/-*^ mice.

### BinJ/JEV_NSW/22-_prME promotes the production of a cross-reactive immune response to other JEV genotypes

**Fig. 4:**
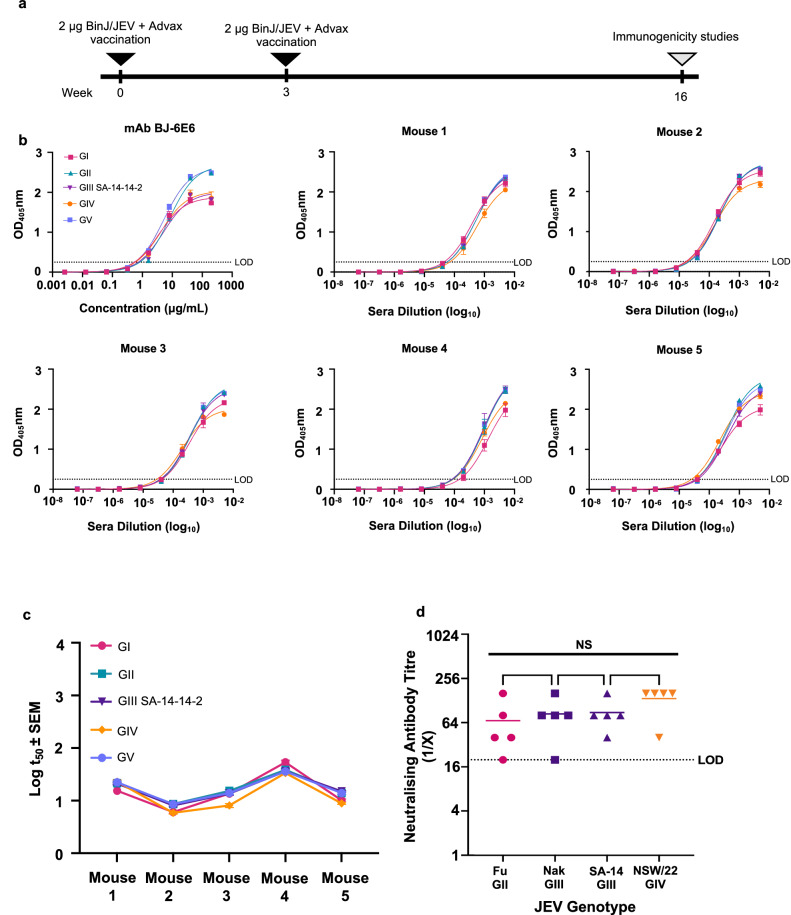
BinJ/JEV_NSW/22_-prME vaccination and immunogenicity studies. **a** Timeline for BinJ/JEV_NSW/22_-prME vaccinations and immunogenicity studies. Serum taken 16 weeks post-vaccination was assessed in immunogenicity studies. **b** Total Ig response to each of the 5 JEV genotypes. **c** Total Ig response to each of the 5 JEV genotypes. Plotted mid-point titres (t_50_) from data displayed in (**b**). **d** Neutralising antibody titres against WT GII (Fu), GIII (Nakayama; SA-14) and GIV (NSW/22). mAb BJ-6E6 was used as a control to ensure sufficient antigen was present. NS not significant.

Five CD1 mice received two s.c. vaccinations 3 weeks apart with 2 µg BinJ/JEV_NSW/22_-prME that was administered with the adjuvant, Advax^TM,65^. The serum from the vaccinated mice was collected 16 weeks post-vaccination (13 weeks-post dose 2) and analysed to assess the anti-JEV antibody response duration and cross-reactivity with other JEV genotypes (Fig. 4a). At 16 weeks post-vaccination, all mice retained a robust total IgG response to all 5 JEV genotypes, with serum anti-JEV IgG end-point titres of 10^4^ to 10^5^ (Fig. 4b). Mid-point IgG titre assessment (t_50_) across the 5 JEV genotypes showed no significant difference (assessed by one-way analysis of variance (ANOVA, α-level 0.05)) in serum antibody binding (Fig. 4c). Similarly, the BinJ/JEV_NSW/22_-prME vaccinated mice developed serum neutralising antibodies against GII, GIII and GIV JEV strains, with no significant difference (assessed by one-way analysis of variance (ANOVA, α-level 0.05)) in neutralisation observed (Fig. 4d). Together these data indicate that the GIV-based BinJ/JEV_NSW/22_-prME chimera elicits a comparable immune response to other JEV genotypes.

### BinJ/JEV_NSW/22-_prME provides partial cross protection against MVEV viremia

**Fig. 5:**
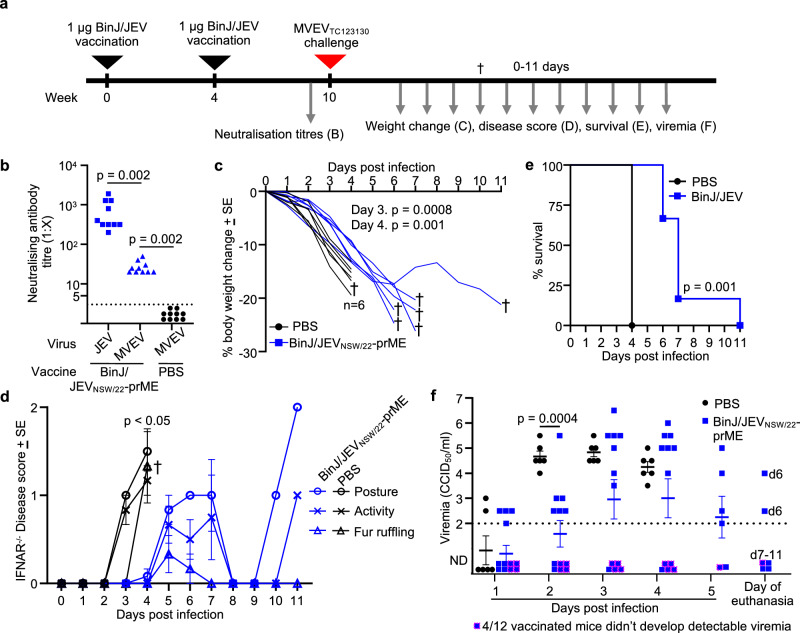
BinJ/JEV_NSW/22-_prME provides partial cross protection against MVEV_TC123130_ in *Ifnar*^*-/-*^ mice. **a** Timeline of BinJ/JEV_NSW/22_-prME vaccinations, MVEV challenge, and sample/data collections. **b** Serum end point neutralizing antibody titers against either JEV_NSW/22_ or MVEV after two vaccinations. Limit of quantification 1 in 5. (Statistics by Wilcoxon matched-pairs signed rank tests). **c** Mean percent mouse body weight change relative to day 0 for each mouse (*n* = 6 per group). Statistics by repeated measure ANOVA for 1 to 4 dpi (*p* = 0.008). **d** Clinical disease scores for posture (circles), activity (crosses) and fur ruffling were monitored in *Ifnar*^*-/-*^ mice. *n* = 6 PBS injected, *n* = 12 BinJ/JEV_NSW/22_-prME vaccinated until 4 dpi, *n* = 6 BinJ/JEV_NSW/22_-prME vaccinated until humane endpoint reached. † Ethically defined end point reached for euthanasia. Statistics by Kolmogorov-Smirnov exact tests (*n* = 6 PBS vaccinated, *n* = 12 BinJ/JEV_NSW/22_-prME vaccinated on 4 dpi), *p* < 0.05 for each disease parameter. **e** % survival; time until mice reached ethically defined end points for euthanasia, *n* = 6 per group. Significance by log rank statistic. **f** Post-challenge viremia in *Ifnar*^*-/-*^ mice determined by TCID_50_ assays. Viremia for individual mice at each time point. Limit of detection was 2 log_10_TCID_50_/ml. ND not detected. In the BinJ/JEV_NSW/22_-prME vaccinated group no viremia was detected on any day in 4/12 mice (blue square with red boarder). Statistics by Kolmogorov-Smirnov exact test on 2 dpi (*n* = 6 PBS injected, *n* = 12 BinJ/JEV_NSW/22_-prME vaccinated).

Given the robust protection of the BinJ/JEV_NSW/22-_prME vaccine against homotypic JEV in both immunocompetent and *Ifnar*^*-/-*^ mouse models, and evidence for the vaccine eliciting comparable humoral responses against other JEV genotypes, we sought to determine if the vaccine would elicit cross-neutralising antibodies and confer cross-protection against MVEV challenge. Female *Ifnar*^*-/-*^ mice received two i.m. vaccinations ≈4 weeks apart with 1 µg of unadjuvanted BinJ/JEV_NSW/22-_prME in 50 µL of PBS, or injected with PBS only (Fig. 5a). Serum end-point neutralising antibody titres were determined after the second vaccination and before MVEV challenge (Fig. 5a). BinJ/JEV_NSW/22-_prME vaccinated mice produced robust neutralising antibody titres against JEV_NSW/22_, which were significantly higher than neutralising antibody titres against MVEV (Fig. 5b, *p* = 0.002). However, all mice had detectable neutralising antibodies against MVEV (Fig. 5b).

PBS-injected *Ifnar*^*-/-*^ mice infected with MVEV showed rapid weight loss (Fig. 5c) and overt clinical disease manifestations (Fig. 5d), with all mice requiring euthanasia on day 4 post-infection (Fig. 5e). Mice vaccinated with BinJ/JEV_NSW/22-_prME had a significant delay in weight loss (Fig. 5c, *p* = 0.008), disease manifestations (Fig. 5d, *p* < 0.05) and mortality (Fig. 5e, *p* = 0.001).

PBS-injected *Ifnar*^*-/-*^ mice infected with MVEV had a robust viremia reaching ~10^5^ TCID_50_/ml on days 2-4 (Fig. 5f). Mice vaccinated with BinJ/JEV_NSW/22-_prME had a delayed viremia, which was significantly lower than unvaccinated mice on day 2 post-infection (Fig. 5f, *p* = 0.0004). Of the 12 BinJ/JEV_NSW/22-_prME vaccinated mice challenged with MVEV, four did not develop a detectable viremia at any timepoint measured (Fig. 5f, blue squares with red boarder). Overall, this suggests that BinJ/JEV_NSW/22_-prME provided partial cross-protection against MVEV.

## Discussion

The 2022 incursion of JEV into the majority of states and territories of mainland Australia was unprecedented and of considerable concern for human and animal health. High levels of rainfall associated with the *La Niña* weather pattern over much of the affected areas during 2021-2023 have been conducive to the spread of mosquito-borne viruses, like JEV, providing ample breeding sites for the main vector (*Culex annulirostris*) and supporting the proliferation of water birds and feral pigs that serve to maintain transmission cycles in rural and peri-urban environments^11^. It is likely that these factors have contributed to the wide distribution of JEV on the Australian continent, with potential for the virus to become established which will continue to pose a significant threat for years to come. Here, we describe the application of the insect-specific flavivirus BinJV platform technology to the development of a BinJ/JEV_NSW/22_ -prME chimeric virus as a JEV vaccine candidate based on the Australian 2022 outbreak strain. We further showed that vaccinated mice produced potent neutralising antibodies against JEV_NSW/22_, and that complete protection from virus challenge in both immunocompetent and *Ifnar*^*-/-*^ mouse models could be elicited. To our knowledge, this is the first GIV JEV vaccine described and represents an important development due to the continued expansion of the less common genotypes and displacement of the once dominant GIII strains^11,47,48^, upon which all current commercial vaccines are based^16^.

JEV continues to cause significant numbers of human infections and fatalities, primarily in the Asian Pacific region, despite efficacious vaccines being widely approved for use. However, replacements for these vaccines are being sought to address safety concerns associated with live attenuated vaccines and manufacturing issues, such as high costs^49,50^. Furthermore, in Australia, no vaccines against JEV are approved for veterinary use, despite pigs and horses being vaccinated in some JEV endemic countries. The chimeric virus created in this study, BinJ/JEV_NSW/22 -_prME, grew to high titres in insect (mosquito) cell culture, but failed to replicate in a panel of vertebrate cell lines, similar to the parental BinJV, which is consistent with our previous studies^27,51^. The ability of this chimeric virus to replicate to high titres in insect cells will be a distinct advantage for large-scale manufacturing and efficient vaccine production. Furthermore, the inability of the BinJ/JEV_NSW/22_ -prME vaccine to replicate in vertebrate cells provides a significant safety aspect to allow propagation of the vaccine in low biocontainment facilities. We are currently generating cell banks of the mosquito cell line to meet regulatory requirements for vaccine production.

In the context of rapid response to emerging outbreaks, authentic chimeric viruses for a new or emerging flavivirus disease can be derived using the BinJV chimeric platform in less than a month, including receipt of synthetic DNA coding for the new flavivirus prM and E genes. This represents a unique and powerful pipeline for rapid response to outbreaks of new flavivirus diseases. Indeed, the BinJ/JEV_NSW/22_ -prME chimera described here was derived 2 weeks after the receipt of the viral RNA, which was used as the template for CPER. Rapid vaccine development is especially important in the context of JEV, as the shifts in the dominant genotype (GIII to GI)^52^ and the emergence of the more divergent genotypes GIV and GV^11,47,48^ have recently been reported. This is particularly relevant for divergent JEV genotypes that exhibit antigenic differences that may affect vaccine efficacy^53^. Therefore, the ability to rapidly adjust the vaccine formulation to the dominant JEV genotype may be of significant benefit, as has also been demonstrated by the regular update and release of new SARS-CoV-2 mRNA vaccines. The rapid pipeline used to generate the BinJ/JEV_NSW/22_ -prME chimera has also been employed to raise and isolate anti-JEV_NSW/22_ prM and E antibodies. The production of two mAbs that are specific to JEV GIV and demonstration through competitive ELISA that they bind discrete epitopes represents the production of powerful new tools in the outbreak scenario. While the newly generated mAbs did not neutralise JEV, new mAb panels have been prepared, providing an opportunity to screen for new and potent therapeutics.

There is a growing body of evidence that antibodies targeting quaternary epitopes, such as the dimer interface of the E protein, are important for virus neutralisation^54^. Although VLPs and inactivated whole virus vaccines have shown promise, it has been previously found that these vaccine candidates are not structurally identical to their infectious flavivirus counterparts^38,39^. The changes are thought to be the result of chemical inactivation methods or the manner in which the E dimers are presented on the surface of the VLPs^27,31,32^. Here, we showed that the purified BinJV/JEV_NSW/22_ -prME virions were antigenically indistinguishable from wild-type JEV particles based on binding affinity profiles of a panel of monoclonal antibodies. This authentic presentation of heterologous prM and E proteins expressed from the backbone of the BinJV genome, corroborates our previous data with other BinJV-based chimeras expressing the prM and E genes of other vertebrate-infecting flaviviruses^27,29,55–57^ and likely contributed to the potent induction of protective neutralising antibodies to JEV_NSW/22_.

Prior to expansion into Australia, GIV was considered one of the least common genotypes of JEV and had not been routinely detected outside of Indonesia^58,59^, thus studies into the characteristics and pathogenicity of isolates of this genotype were lacking. However, the fatal infection of an Australian person with GIV JEV, acquired from Bali in 2019^60^ in combination with the isolation of the same strain from pigs and mosquitoes in Bali^61,62^ indicates a strong foothold of this genotype within the region. Neutralising antibodies induced by the JEV GIII-based vaccines approved for use in humans and other animals may produce lower neutralising titres against JEV_NSW/22_^43,63,64^_,_ prompting our development of a vaccine based on the Australian 2022 outbreak strain. Administration of two 1 µg doses of BinJ/JEV_NSW/22_-prME, without adjuvant provided complete protection against the homotypic virus infection in two mouse models of disease, protecting against overt clinical disease manifectations and viraemia. Vaccination of mice with BinJ/JEV_NSW/22_-prME also elicited a cross-reactive and cross-neutralising antibody response against JEV of other genotypes, with additional studies planned to confirm protection afforded by the vaccine against lethal challenge with JEV of other genotypes.

MVEV cases in humans are largely confined to Northern Australia^25^, with rare outbreaks occurring in south-eastern Australia. The first human case of MVEV in the southern Australian state of Victoria since 1974 was confirmed during the 2022/2023 mosquito season^65^ and linked to an unprecedented surge in cases. Studies in horses and mice show that MVEV cross-neutralising antibodies and partial protection can be elicited via vaccination with various JEV vaccines, although not consistently for all animals^66^ and typically at substantially lower neutralisation titres in comparison to those against JEV^67,68^. Consistent with these reported observations, our BinJ/JEV_NSW/22_-prME vaccine elicited a cross-neutralising antibody response against MVEV in all mice vaccinated, however, the neutralising antibody titres against MVEV were significantly lower than those generated against JEV. BinJ/JEV_NSW/22_-prME did not elicit antibody dependent enhancement (ADE) of MVEV infection, which has been suggested as a risk in low dose vaccination settings in mice^67–69^. How useful such reports of in vitro or in vivo ADE for JEV and MVEV are at predicting what happens in large animal hosts is unclear, with a JEV vaccine shown to produce antibodies with ADE activity in vitro but was protective in pigs^70^.

This report describes preclinical efficacy data for a BinJ/JEV-prME vaccine based on the GIV Australian 2022 outbreak strain in mouse models of JEV infection and disease and provides additional support for the utility of the BinJV technology for flavivirus vaccine development. To our knowledge, the studies herein report the first efficacy studies for a GIV-based JEV vaccine and provide valuable support for the progression of the vaccine towards veterinary applications.

## Methods

### Ethics statement and approvals

All mouse work was conducted in accordance with the “Australian code for the care and use of animals for scientific purposes” as defined by the National Health and Medical Research Council of Australia^71^. Animal experiments performed at the University of Queensland were approved by the University of Queensland Animal Ethics Committee (AEC permit numbers 2021/AE000626, AIBN/SCMB/118/20). Mouse work performed at QIMR-Berghofer MRI was approved by the QIMR-Berghofer MRI animal ethics committee (P3746, A2108-612). Breeding and use of genetically modified mice was approved under a Notifiable Low Risk Dealing (NLRD) Identifier: NLRD_Suhrbier_Oct2020: NLRD 1.1(a). All JEV work was conducted in a biosafety level-3 (BSL3) facility at the QIMR-Berghofer MRI (Australian Department of Agriculture, Fisheries and Forestry certification Q2326 and Office of Gene Technology Regulator certification 3445) and the University of Queensland (Australian Department of Agriculture, Fisheries and Forestry certification Q1711). The in vitro and in vivo work performed with JEV were approved under the Queensland Biosecurity Act (Permit number: PRID000916). Tissue collection for the isolation of equine dermal fibroblast cells was approved by the UQ Animal Ethics Committee (permit number 2021/AE000454).

### Cell lines and culture

For the studies performed at UQ, C6/36 cells (*Aedes albopictus*, ATCC CRL-1660) were maintained in Rosewell Park Memorial Institute 1640 (RPMI 1640) medium, supplemented with 5% fetal bovine serum (FBS) at 28 °C. The vertebrate cell lines used in this study were maintained at 37 °C with 5% CO_2_. Vero 76 cells (*Cercopithecus aethiops*, African green monkey kidney, ATCC CRL-1587) and BSR cells (*Mesocricetus auratus*, baby hamster kidney) were grown in Dulbecco’s modified Eagle medium (DMEM) supplemented with 5% FBS. All cell culture media were supplemented with penicillin (50 U/mL), streptomycin (50 μg/mL) and 2 mM L-glutamine (PSG). Vero E6 cells (*Cercopithecus aethiops*, African green monkey kidney, ATCC CRL-1586) were used for all QIMR-based experiments, and were maintained as described for Vero 76.

Equine dermal fibroblast primary cells were isolated using the explant method. Clinically well horses were euthanised via i.v. injection of Xylazine (1 mg/kg) and Pentobarbital (120 mg/kg). Within 2 h of euthanasia, skin tissues were taken from the peri-orbital or inguinal area and immediately transported to the laboratory on wet-ice in DMEM, supplemented with 10% horse serum (HS) and washed in PBS for 15 min. Subsequently tissues were cut into ~0.5 cm^3^ pieces and digested in a Dispase II/PBS solution (1.8 U/mg/mL) before separating the epidermal and dermal layers. Dermal explants were placed in 6-well tissue culture plates and cultured in DMEM containing 10% HS and PSG in a humidified incubator with 5% CO_2_ at 37 °C for 48 h undisturbed. Dermal explants were removed after 96 h, before washing adherent cells with PBS and maintaining cultures in DMEM with 10% HS and PSG. Visual inspection under the microscope was performed every 24 h to check for microbial or fungal contamination, with any contaminated cultures terminated immediately with 70% ethanol. Once 80% confluency was reached, the cells were transferred to a T25 culture flask and designated as “passage 1”. Cells from passage 3 were cryopreserved, while experiments were performed using thawed cells between passage 4 and 7 in DMEM supplemented with 10% FBS and PSG.

### Virus isolates and culture

The JEV_NSW/22_ isolate (O-0883/NSW/22) was obtained from an infected porcine neonate and characterised as genotype IV (Genbank: OP904182). A stock of this isolate (0909) was generated by passage in cell culture using the C6/36 cells. The following additional virus isolates were used herein: JEV GII FU strain (Genbank AF217620.1)^72^, JEV GIII Nakayama strain (Genbank: EF571853), JEV GIII SA14 (Genbank: U14163); MVEV 1-51 (Genbank AF161266), WNV_KUN_ NSW2011 strain (Genbank: JN887352)^73^ was obtained from Dr Peter Kirkland (Elizabeth Macarthur Agriculture Institute) and MVEV_TC123130_ (GenBank: JN119814.1) was obtained from the “Doherty Virus Collection” currently held at QIMR Berghofer MRI. Virus stocks for the mouse studies were assessed for the presence of *Mycoplasma* spp. and endotoxin^74,75^.

Virus stocks were generated by infecting Vero 76 (UQ) or Vero E6 (QIMR) or C6/36 cell monolayers with virus at a multiplicity of infection (MOI) of 0.1 and incubating at 28 °C (C6/36 cells) or 37 °C with 5% CO_2_ (Vero 76 or E6 cells) for 1 h. Following this, inoculum was removed and replaced with fresh growth media containing 2% FBS and incubated at the appropriate temperature for 5–7 days post-infection (dpi), before harvesting by centrifuging at 3000 rpm (Jouan CR132 centrifuge), 4 °C for 10 min. The clarified supernatant was then supplemented with additional FBS to increase the total concentration to 10%, aliquoted and stored at −80 °C until required. Virus stock titres were calculated based on the 50% tissue culture infectious dose (TCID_50_)^76^.

For titration of the JEV and MVEV stocks used in mouse studies, Vero E6 cells (ECACC Vero C1008, Sigma Aldridge, St. Louis, MO, USA) were plated into 96 well flat bottom plates at 2 × 10^4^ cells per well in 100 µl of medium. For tissue titrations, tissues were homogenized in tubes, each containing 4 ceramic beads, twice at 6000 x rpm for 15 s, followed by centrifugation twice at 21,000 x g for 5 min before 5 fold serial dilutions in 100 µl RPMI 1640 supplemented with 2% FBS. For cell culture supernatant or mouse serum, 10 fold serial dilutions were performed in 100 µl RPMI 1640 supplemented with 2% FBS. A volume of 100 µl of serially diluted samples were added to each well of 96 well plate containing Vero E6 cells and the plates cultured for 5–7 days at 37 °C and 5% CO_2_. The endpoint dilution where cytopathic effects were evident was recorded and the virus titre (TCID_50_) was calculated by the method of Spearman and Karber^77,78^.

### Circular polymerase extension reaction to construct BinJ/JEV_NSW/22-_prME

Chimeric infectious DNA constructs between BinJV (Genbank Accession number MG587038) and JEV_NSW/22_ were generated by circular polymerase extension reaction (CPER) as previously described^27^. Briefly, BinJV and JEV_NSW/22_ viral RNA were converted to cDNA using SuperScript IV reverse transcriptase (ThermoFisher Scientific) as per the manufacturer’s instructions. The cDNAs were then used as templates for a set of primer pairs to produce six overlapping dsDNA fragments covering the BinJV backbone and JEV_NSW/22_ prME insert. For each CPER assembly, 0.1 pmol of each viral cDNA fragment was added to a Q5 PCR (NEB) as per the manufacturer’s instructions. Thermal cycling conditions were as follows: [98 °C, 2 min]_x1_; [98 °C/ 30 sec; 55 °C/ 30 s; 72 °C/ 6 min]_x2_; [98 °C/ 30 s; 55 °C/ 30 s; 72 °C/ 6 min]_x10_; [14 °C] _hold_. The entire CPER was transfected into C6/36 cell monolayers using TransIT-LT1 (Mirus) as per the manufacturer’s instructions, and the passage 0 (P_0_) cell culture supernatants were harvested 7 days post-transfection and stored at −80°C.

To confirm the presence of replicating virus following CPER, preliminary analysis was performed by trypsinising P0 C6/36 monolayers and seeding the cells onto glass coverslips. The cells were incubated for a further 24 h prior to being fixed in 100% ice-cold acetone. Immunofluorescence assays (IFA) were then performed as previously described, using the pan-flavivirus reactive NS1 monoclonal antibody (mAb) 4G4 (diluted 1/10)^27,51^.

### Production of chimeras for antigenic analysis

For antigenic analysis (defined below) using mAbs, a range of BinJV-based chimeras were produced using the same CPER strategy as for the BinJ/JEV_NSW/22_-prME chimera, as previously published^27^. Overlapping dsDNA prM/E inserts for the following JEV genotypes were used: GI (GZ56 isolate, HM366552.1), GII (FU isolate, AF217620.1), GIII (vaccine SA-14-14-2 isolate, JN604986), GIII (p3 strain, U47032.1) and GV (XZ0934 isolate, JF915894.1).

### In vitro host range assessment

To ensure the host range restriction of BinJ/JEV_NSW/22-_prME in vitro, vertebrate cell infection assays were performed as previously detailed^27,51^. Briefly, monolayers of C6/36 cells and a panel of vertebrate cell lines (Vero 76, BSR and equine dermal fibroblasts) were grown on glass coverslips before inoculating with virus at an MOI of 1 (C6/36, Vero 76 and BSR) or an MOI of 10 (equine dermal fibroblasts). Following incubation for 1 h with rocking at room temperature, the inoculum was removed, and the coverslips washed three times with sterile PBS before adding appropriate growth medium back onto the coverslips. The coverslips were cultured for 5 dpi (C6/36, Vero 76 and BSR) or 7 dpi (equine dermal fibroblasts), before harvesting the supernatant and storing at −80 °C. The coverslips were then fixed in 100% ice-cold acetone, before assessing for the presence of viral replication by an indirect IFA^27,51^.

### BinJ/JEV_NSW/22-_prME vaccine production

The production of BinJV-based chimeric vaccines for several pathogenic flaviviruses has been previously described^27,29,31^. Sub-confluent monolayers of C6/36 cells were infected with BinJ/JEV_NSW/22-_prME at an MOI of 0.1. Supernatant was collected at 3-, 6-, 8- and 11-dpi. The virus culture supernatant was clarified by centrifugation at 3000 rpm (Jouan CR132 centrifuge) for 30 min at 4 °C and filtered through a 0.22 μM filter. After each collection, cells were replenished with fresh RPMI containing 2% FBS. The BinJ/JEV_NSW/22-_prME virions were precipitated from the harvested culture supernatant via addition of polyethylene glycol 8000 to a final concentration of 8% and slow stirring overnight at 4 °C. The virus was pelleted at 28,000 rpm (Beckman Coulter JLA 10.500 rotor) for 1.5 hr at 4 °C, before ultracentrifugation through a sucrose cushion and potassium tartrate gradient as described previously^27,31^. Purified virus was collected, and buffer exchanged into sterile PBS using a 30 kDa molecular weight cut-off Amicon filter and stored at 4 °C. Purified virus was quantified using the amount of E protein against a BSA standard following SDS-PAGE, total protein staining (SYPRO Ruby, Invitrogen) and analysis using Image J^27,29,31^.

### Negative staining electron microscopy

Purified BinJ/JEV_NSW/22-_prME was diluted to 400 μg/mL in PBS, before adsorbing onto carbon-coated grids (ProSciTech, Thuringowa, QLD, Australia) for 2 min and glow-discharged for 5 sec in 25 mA. The grids were blotted and washed three times in water and stained twice with a 2% uranyl acetate solution, with blotting in between. The grids were air-dried and imaged using a Hitachi HT7700 microscope operated at 120 Kv.

### Next-generation sequencing

To confirm the sequence identity of the vaccine (BinJ/JEV_NSW/22_-prME), deep sequencing was performed on RNA extracted from the concentrated virions produced for the vaccine described above. The RNA was extracted using the Macherey-Nagal Nucleospin RNA Virus kit as per the manufacturer’s instructions, with the following modifications: carrier RNA was omitted from the lysis buffer^79^; 20 µL of Proteinase K (20 mg/mL) was added to the initial lysis mixture and incubated at 70 °C for 5 min; and an in-column DNAse I disgestion step was performed after the first wash step by adding 5 µL DNAse I (NEB). The purified RNA was sequenced at the Australia Genome Research Facility (AGRF, Melbourne, Australia) using the NovaSeq SP Illumina platform, generating 2 ×150-base paired reads. The viral genome was assembled using the expected sequence as a scaffold and Geneious Prime software (version 2023.1.1).

### Monoclonal antibody production and antigenic analysis

To generate mAbs reactive to the Australian outbreak strain of JEV, BALB/c mice (Animal Resources Centre, Murdoch, Western Australia) were immunised with purified BinJ/JEV_NSW/22-_prME adjuvanted with Advax^TM^ (Vaxine Pty Ltd., Adelaide, Australia), as previously described^27^ or with baculovirus-expressed JEV prM/E antigen derived from a 1995 Australian JEV GII isolate^4^, using previously described protocols^80^, and the JEV FU strain prM/E insert. Mice were immunised with 2 doses of the antigen via the s.c. route 2 weeks apart and the mice were bled 2 weeks after the second dose via the tail vein. Seroconversion was verified by fixed-cell ELISA. The mice were admistered a final boost via the i.v. route without adjuvant >10 weeks following the second dose and the spleens harvested 4 days later following euthanasia by cervical dislocation. Fusion of the spleen cells with NS0 (European Collection of Cell Cultures) or X63-Ag8.653 myeloma cells was performed as previously described^81–83^. Hybridomas secreting JEV-reactive mAbs were identified by fixed-cell ELISA as previously described^36,82^. The mAbs were further analysed for reactivity in Western blot and fixed cell-ELISA for cross-reactivity to additional JEV genotypes using BinJV-based JEV GI-V chimeras described above and other closely related local wild type flaviviruses (WNV_KUN_, MVEV), with incubation periods being performed at 37 °C using methods previously detailed^36^. The isotype of each mAb was determined using mouse monoclonal antibody isotyping reagents (Sigma-Aldrich, St Louis, Missouri, United States) as per the manufacturer’s instructions. The neutralisation capabilities of each mAb was assessed using BinJ/JEV_NSW/22_-prME and methods previously detailed using C6/36 cells^81^.

To assess the antigenicity of our new chimeric virus, the reactivity profile of mAbs to BinJ/JEV_NSW/22-_prME and the parental wildtype (WT) JEV strain was determined using fixed-cell ELISA. For apparent dissociation analyses (K_d_), the mAbs were purified by protein G or IgM affinity chromatography (Hi Trap, Cytiva), before end-point titration using a serial 5-fold dilution series starting at a concentration of 100-200 ug/mL and performing an ELISA as previously described^27^. Apparent affinity (K_d_) was established by non-linear regression with a one site-specific binding model using Graphpad Prism 9 software.

### Competitive ELISA

ELISA plates (Greiner high binding) were coated with 100 ng/well of purified BinJ/JEV_NSW/22-_prME and incubated at 4 °C overnight in ELISA coating buffer (50 mM NaHCO_3_, 50 mM Na_2_CO_3_; pH 9.6). After washing with phosphate buffered saline/0.05% Tween 20 (PBST), the plates were blocked with ELISA blocking buffer (0.05 M Tris-HCl [pH 8.0], 1 mM EDTA, 0.15 M NaCl, 0.05% [vol/vol] Tween 20, 0.2% [wt/vol] casein). A saturating concentration of purified unlabeled mAbs (1 µg/mL) was added in triplicate wells, followed by incubation at 37 °C for 1 h. Without removing the unlabeled mAbs (all IgG isotype), a predetermined nonsaturating concentration of mAb JV-4H12 (IgM) was added, and the plates incubated for a further 1 h at 37 °C. After a washing step with PBST, bound mAb JV-4H12 was detected with 1 μg/mL HRP-conjugated goat anti-mouse IgM (Invitrogen, Catalog # 31440) for 1 h. After a final wash, substrate solution [1 mM 2,2-azino-bis(3-ethylbenzthiazoline-6-sulfonic acid) (ABTS) and 3 mM H_2_O_2_ in a buffer prepared by mixing 0.1 M citric acid with 0.2 M Na_2_HPO_4_ to give a pH of 4.2] was added to each well, and the plates were incubated in the dark at room temperature for 1 h. The absorbance was measured at 405 nm.

### Mouse vaccination and JEV_NSW/22_ challenge

Female C57BL/6J (≈6-week old, *n* = 10 per group) mice were purchased from the Animal Resources Centre (Canning Vale, Western Australia, Australia) and *Ifnar*^-/-^ (≈14-week old, *n* = 10 per group) mice on a C57BL/6J background^71^ were bred in-house^44^. Mice were vaccinated with purified BinJ/JEV_NSW/22-_prME as previously described, without adjuvant^30,44^. Briefly, vaccines were administered intramuscularly (i.m.) into both quadriceps muscles of anaesthetised mice with 50 μl per muscle using a 27 G needle. Mice were vaccinated twice with either 1 μg of BinJ/JEV_NSW/22-_prME or injected with PBS as a mock-vaccination, separated by ≈4 weeks. Serum neutralising antibody titres were determined just prior to the second dose, and ≈4 weeks after the second dose.

Six weeks after the second vaccination, mice were challenged with 5×10^3^ TCID_50_ diluted to a total volume of 100 μl of JEV_NSW/22_ (GenBank: OP904182.1) subcutaneously (s.c.) in the base of the tail. Mice were monitored using a score card system that provided a score (1 = mild, 2 = moderate, 3 = severe) for posture, activity, fur ruffling and weight loss- clinical manifestations of disease in the JEV mouse model^43^. Mild weight loss was 10–15%, moderate weight loss was 15–20%, and severe weight loss was >20%. Mice reaching a score of 2 or more for any two disease manifestations, or a score of 3 in any one disease manifestation were euthanised using carbon dioxide. Blood was collected at the indicated timepoints via lateral tail vein bleed into serum separator tubes and the aliquoted serum was stored at -80 °C. Mice were euthanised on days 2, 4 and 10 post-challenge, and brain and spleen tissues collected. Tissue and serum titres were determined by TCID_50_ assays, as described^32,84,85^. Briefly, samples were titrated in duplicate starting with a 1 in 10 dilution followed by 10-fold serial dilutions on C6/36 cells (5-day culture). Subsequently, parallel well-to-well transfer of supernatants into 96-well plates containing Vero E6 cells was carried out. After 5 days of culture, cytopathic effects were observed.

### End-point neutralisation titre determination

The JEV end-point neutralising antibody titres were determined essentially as described^84^ against JEV_NSW/22_ by incubating 2-fold serial dilutions of heat-inactivated (56 °C, 30 min) mouse serum (in duplicate, 50 μl) with 150 TCID_50_ of virus (50 μl) for 1 h before adding 100 μl Vero E6 cells diluted to 10^4^ cells/well in RPMI 1640 media supplemented with 2% FBS. Starting dilution was 1:10. After 7 days, neutralisation titres were expressed as the highest dilution where protection against cytopathic effects was observed by inverted microscope.

### Quantitative RT-PCR of mouse brains and spleen

Quantitative RT-PCR (qRT-PCR) was performed using iTaq Universal Probes One-Step Kit (Bio-Rad, California, USA) as per manufacturers instructions and using JEV_NSW/22_-specific primers (Integrated DNA Technologies): forward 5’- TGGGCCTTCTGGTGATGTT -3’ and reverse 5’- CCCAAGCATCAGCACAAG -3’, and probe 5’ – /56-FAM/AAGAGGTGG/ZEN/ACGGCCAGATTGAC/3IABkFQ/ -3’, with normalization to the housekeeping gene, mouse *Rpl13a* forward primer 5′- GAGGTCGGGTGGAAGTACCA -3′, reverse primer 5′- TGCATCTTGGCCTTTTCCTT -3′ as described^86^. Reactions were performed using the CFX 96 touch PCR detection system (Bio-Rad, California, USA) and data analysed using Biorad CFX Real Time Analysis software.

### Mouse vaccination and MVEV challenge experiment

*Ifnar*^-/-^ (≈14-week-old) mice on a C57BL/6J background were vaccinated with purified BinJ/JEV_NSW/22_-prME as described above (Section: Mouse Vaccination and JEV_NSW/22_ challenge). Six weeks after the second vaccination, mice were challenged s.c. with 5 × 10^3^ TCID_50_ of MVEV_TC123130_ (GenBank: JN119814.1) diluted to a total volume of 100 µl. Mice were monitored for disease using a scorecard system as described above. PBS injected mice were euthanised on day 4 post challenge due to reaching terminal disease criteria (see above). One group of BinJ/JEV_NSW/22-_prME vaccinated mice was also culled on day 4 post challenge to collect samples at the matching timepoint. The remaining group of BinJ/JEV_NSW/22_-prME vaccinated mice was monitored for survival. Blood was collected at the indicated timepoints via lateral tail vein bleed into serum separator tubes and the aliquoted serum was stored at −80 °C. Viremia was determined by TCID_50_ assay as described above for JEV_NSW/22_.

### Mouse vaccination for immune response cross-reactivity analysis

Six to eight week-old female CD1 mice were immunised twice with 2 µg BinJ/JEV_NSW/22_-prME, 3 weeks apart via the subcutaneous route at the base of the tail using a 26 G needle, with a maximum volume of 100 µl. The vaccine was prepared within 30 min of vaccination by combining virus with the adjuvant Advax^TM^ (Vaxine Pty Ltd., Adelaide, Australia, 1 mg/mouse) in PBS. Serum was procured via tailbleed and assessed 16 weeks post dose 1 (13 weeks post dose 2). Total IgG responses against each of the different JEV genotypes was assessed by fixed-cell ELISA, whereby the serum from each mouse was titrated out on C6/36 cell monolayers that had been infected with each of the chimeras described above. Serum neutralisation titres were determined by microneutralisation assays as previously described^31^ using Vero 76 cells and an incubation period of 3 days and the WT JEV strains FU (GII), Nakayama (GIII), SA-14 (GIII) and NSW/22 (GIV), and between 65-269 infectious particles, as determined by back-titration^31^.

### Statistics

The *t*-test was used when the differences in variances was <4 fold, skewness was >−2 and kurtosis was <2. Otherwise, the non-parametric Kolmogorov-Smirnov exact test was used (GraphPad Prism 10). For Fig. 1c, a one-way analysis of variance (ANOVA) was performed for multiple comparison analysis with the α-level set at 0.05 with a Tukey’s post test, while Fig. 2f was analysed by Pearson correlations (Graphpad Prism 10). Repeated measure ANOVA tests were performed using IMB SPSS Statstics for Windows, Version 19.0 (IBM Corp., Armonk, NY, USA).

### Reporting summary

Further information on research design is available in the Nature Research Reporting Summary linked to this article.

### Supplementary information

Supplementary Material

Reporting Summary

## Data Availability

The authors declare that all data supporting the findings of this study are available within the paper and its supplementary information files.
