# Supplementary material for: A chimeric vaccine derived from Australian genotype IV Japanese encephalitis virus protects mice from lethal challenge
Source: NPJ Vaccines. 2024 Jul 31;9:134. doi: 10.1038/s41541-024-00903-2 (PMC11291493; doi:10.1038/s41541-024-00903-2)
Supplement: Supplementary file 1 — Supplementary Material [file 41541_2024_903_MOESM1_ESM.pdf]

Supplementary Figures and Tables

Supplementary Table 1: Deep sequencing analysis of recovered BinJ/$JEV_{NSW/22}$-prME chimeric virus

Nucleotide Changes		Amino acid change	
Position (gene)	Nucleotide change	Position (gene)	Amino acid change
480 (prM)*	C → T*	No changes	No changes
4, 626 (NS3)	A → C	No changes	No changes
9, 603 (NS5)	C → A	No changes	No changes
10, 095 (NS5)	G → T	No changes	No changes

*Intentional silent mutation introduced

Supplementary Table 2: Reactivity of mAbs to JEV GI-V and other members of the JEV serocomplex in fixed-cell ELISA

mAb	Target*	Neutralisation#	Isotype	mAb Reactivity in ELISA^§								Reference
				JEV genotype						$MVEV_{1-51}$	WNV_{KUN} (NSW2011)	
				GI_{GZ56}†	GII_{Fu}†	$GIII_{SA-14-14-2}$†	$GIII_{p3}$†	$GIV_{NSW/22}$	GV_{ZX0934}†			
4G2	EDII	ND	IgG2a	+	+	+	+	+	+	+	+	[88]
BJ-6E6	EDII	ND	IgG1	+	+	+	+	+	+	+	+	[27]
6B6C-1	EDII	ND	IgG2a	+	+	+	+	+	+	+	+	[89]
M2-8E7	EDIII	<10	IgG2a	+	+	+	+	+	+	+	-	[59]
989¶	prM, Eα	<10	IgG2a	+	+	+	+	+	+	-	-	[61]
BJ-1E1	EDII	ND	IgG1	ND	ND	ND	ND	+	ND	+	+	[27]
P3H8	EDII	ND	IgM	ND	ND	ND	ND	+	ND	+	+	[27]
M2-1E7	ND	ND	IgG2a	ND	ND	ND	ND	+	ND	+	+	[59]
2E9	E	ND	IgM, IgG1	+	+	+	+	+	+	+	+	This study
4D10	prM, E	ND	IgM	+	+	+	+	+	-	-	-	This study
JV-2G7	E	<10	IgM	+	+	+	+	+	+	+	+	This study
JV-4H12	EDIII	<10	IgM	-	-	-	-	+	-	-	-	This study
JV-7F11	E	<10	IgG1	-	-	-	-	+	-	-	-	This study

*Western blot analysis whereby prME = 75 KDa protein; E = 50 KDa protein; prM = 25 KDa protein. #Neutralisation titre taken at the highest dilution that inhibits all virus replication, neutralisation assay performed using $JEV_{NSW/22.}$ ^ Cut-off for a positive result was 2 x OD of the same dilution of mAb on mock cells. §Strain used indicated in subscript text. †Antigen provided as a BinJV chimera e.g. BinJ/JEV G1-prME. ND = not done. ¶ Originally described as mAb 995. αSee Supplementary Figure 1 for target confirmation.

Supplementary Table 3: K_d values for binding of mAbs to BinJ/JEV-prME genotypes I-V

mAb	Virus*							
	JEV[#]						$WNV_{KUN\ (NSW2011)}$	$MVEV_{1-51}$
	GI_{GZ56}	GII_{Fu}	$GIII_{SA14142}$	$GIII_{p3}$	$GIV_{NSW/22}$	GV_{ZX0934}		
4G2	0.31±0.051	0.345±0.028	13.216±3.226	1.54±0.155	0.367±0.049	0.475±0.060	0.64±0.093	1.304±0.548
BJ-6E6	0.14±0.02	0.245±0.021	0.093±0.016	0.154±0.018	0.1±0.014	0.09±0.009	0.151±0.021	0.119±0.03
6B6C-1	0.236±0.025	0.183±0.017	0.424±0.036	0.695±0.109	0.278±0.042	0.366±0.049	0.241±0.035	0.586±0.137
M2-8E7	0.2608±0.0149	0.246±0.017	0.281±0.016	>100	0.261±0.019	0.206±0.012	>100	0.337±0.102
989	1.665±0.093	2.239±0.213	1.539±0.079	1.823±0.144	2.039±0.116	1.249±0.085	>100	>100
JV-4H12	>100	>100	>100	>100	3.175±1.097	>100	>100	>100
JV-7F11	>100	43.073±27.782	>100	>100	0.162±0.042	>100	33.342±46.845	86.161±163.925
2E9	7.594±1.201	7.621±3.642	7.622±1.162	14.802±2.546	5.754±3.269	22.646±5.592	11.013±1.751	89.91±26.856
JV-2G7	2.766±0.261	2.423±0.466	3.326±0.248	4.181±0.521	2.567±0.139	2.217±0.150	3.563±0.368	±

* Strain used indicated in subscript text. [#]Antigen provided as a BinJV chimera expressing the prM and E genes

Key
kD < 0.1
kD < 1
kD < 1.5
kD < 2
kD < 10
kD < 15
kD < 50
kD < 99
kD < 100

Supplementary Table 4: K_d values for binding of mAbs to BinJ/JEV_{AU22}-prME and $JEV_{NSW/22}$. List of mAbs and raw data used to generate Fig 2F.

mAb	Viral Target	$JEV_{NSW/22}$		BinJ/JEV_{AU22}-prME		Reference
		Kd	SE	Kd	SE	
4G2	EDII	0.526	0.092	0.367	0.049	[88]
BJ-6E6	EDII	0.091	0.017	0.096	0.014	[27]
6B6C-1	EDII	0.498	0.083	0.278	0.042	[89]
M2-8E7	EDIII	0.401	0.088	0.260	0.019	[59]
989[#]	prM, E	1.439	0.326	2.039	0.115	[61]
JV-4H12	EDIII	1.882	0.644	3.175	1.097	This study
JV-7F11	E	0.283	0.043	0.162	0.042	This study
2E9	E	5.734	1.231	5.754	3.269	This study
JV-2G7	E	2.899	0.553	2.567	0.139	This study
BJ-1E1	EDII	0.017	0.002	0.099	0.002	[27]
P3H8	EDII	0.373	0.109	0.378	0.135	[27]
M2-1E7	ND	0.006	0.001	0.005	0.001	[59]

[#]originally described as mAb 995

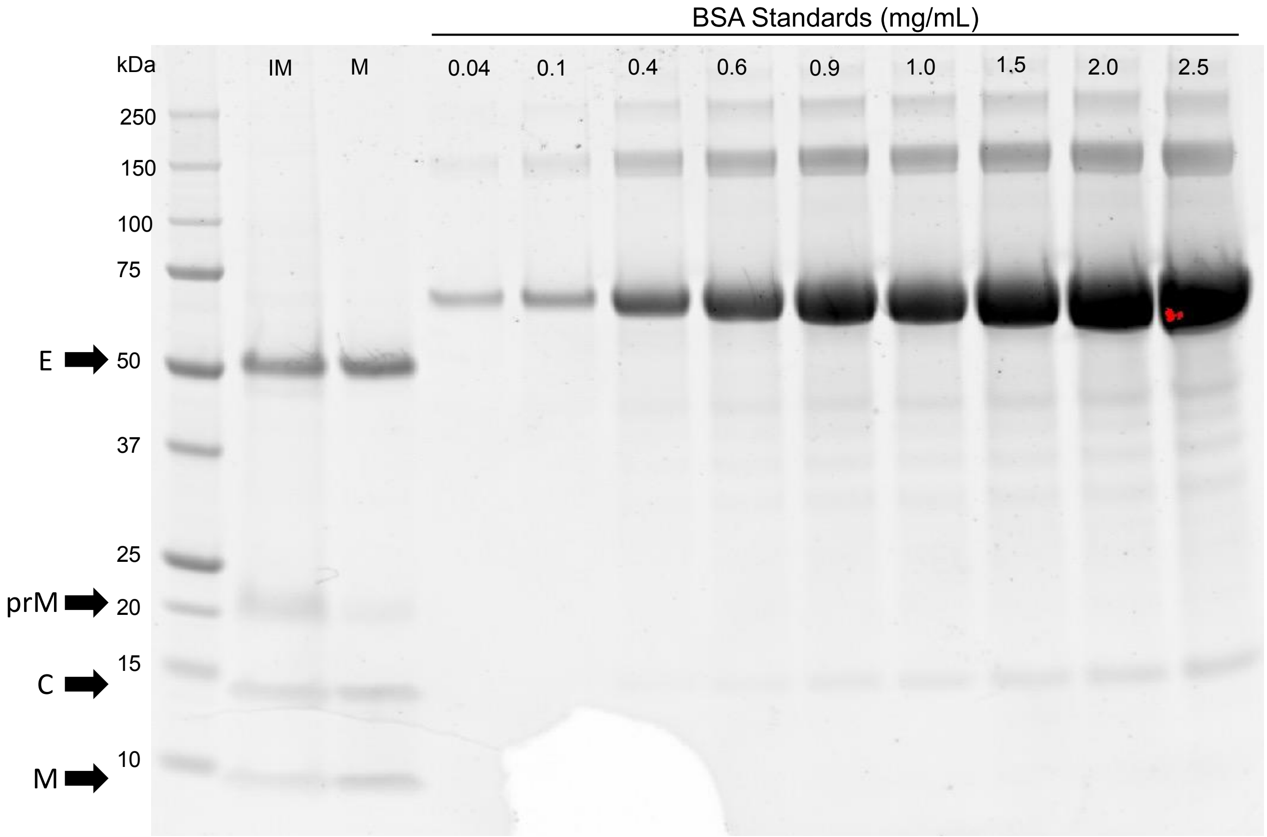

Supplementary Figure 1. Quantification of BinJ/JEV$_{NSW/22}$-prME vaccine antigen. BinJ/JEV$_{NSW/22}$-prME was purified via a potassium tartrate gradient and analysed by SDS-PAGE (4-12%) and sypro Ruby staining. The vaccine antigen was quantified against known BSA standards in Image J. Raw SDS-PAGE of Fig 2B. IM = immature virions and M = mature virions.

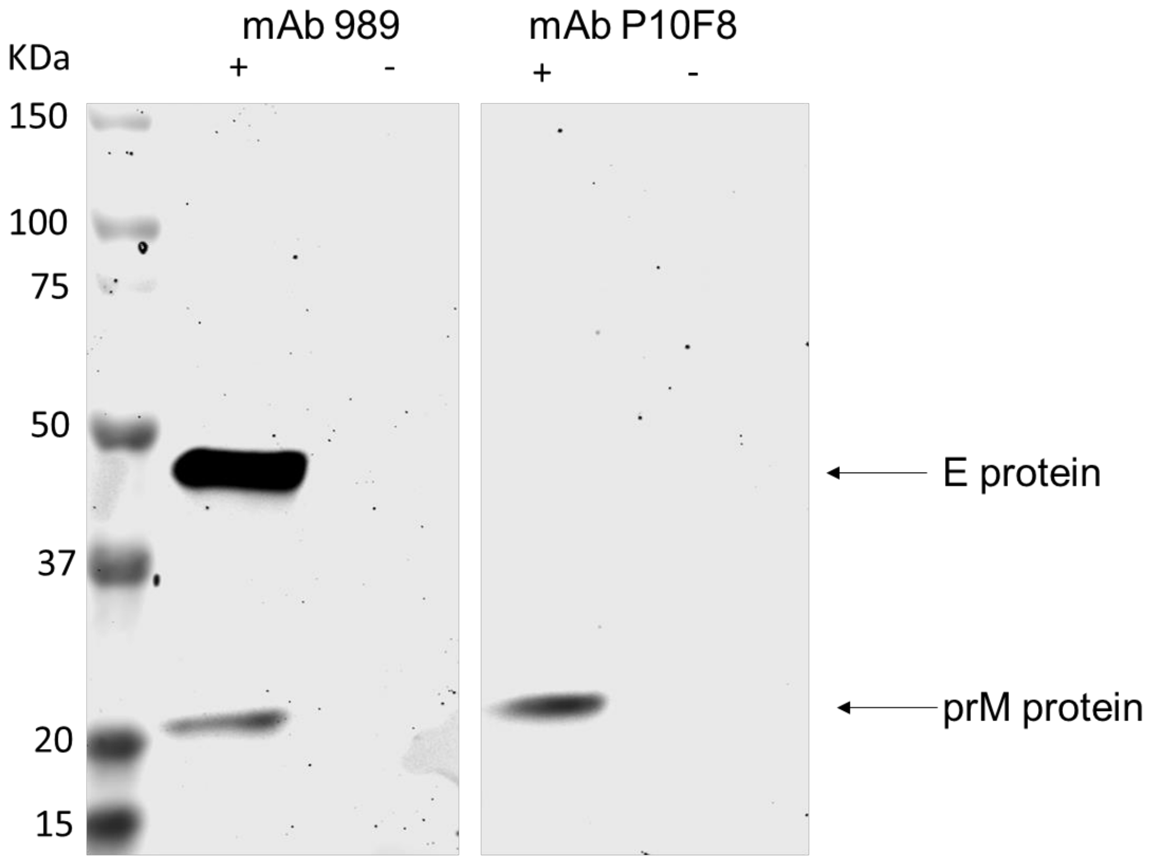

Supplementary Figure 2: Western blot characterisation of JEV mAb 989. To confirm the viral protein target of JEV mAb 989, Western blot analysis using native lysates of C6/36 cells infected with BinJV/JEV$_{AU/22}$-prME (for mAb 989, left panel) or WNV$_{KUN}$ (for mAb P10F8, right panel) or mock infected. The primary reactivity of each mAb is indicated. P10F8 is known to bind WNV$_{KUN}$ prM and was included as a positive control. "+" indicates virus-infected cell lysate; "-"indicates mock-infected lysate

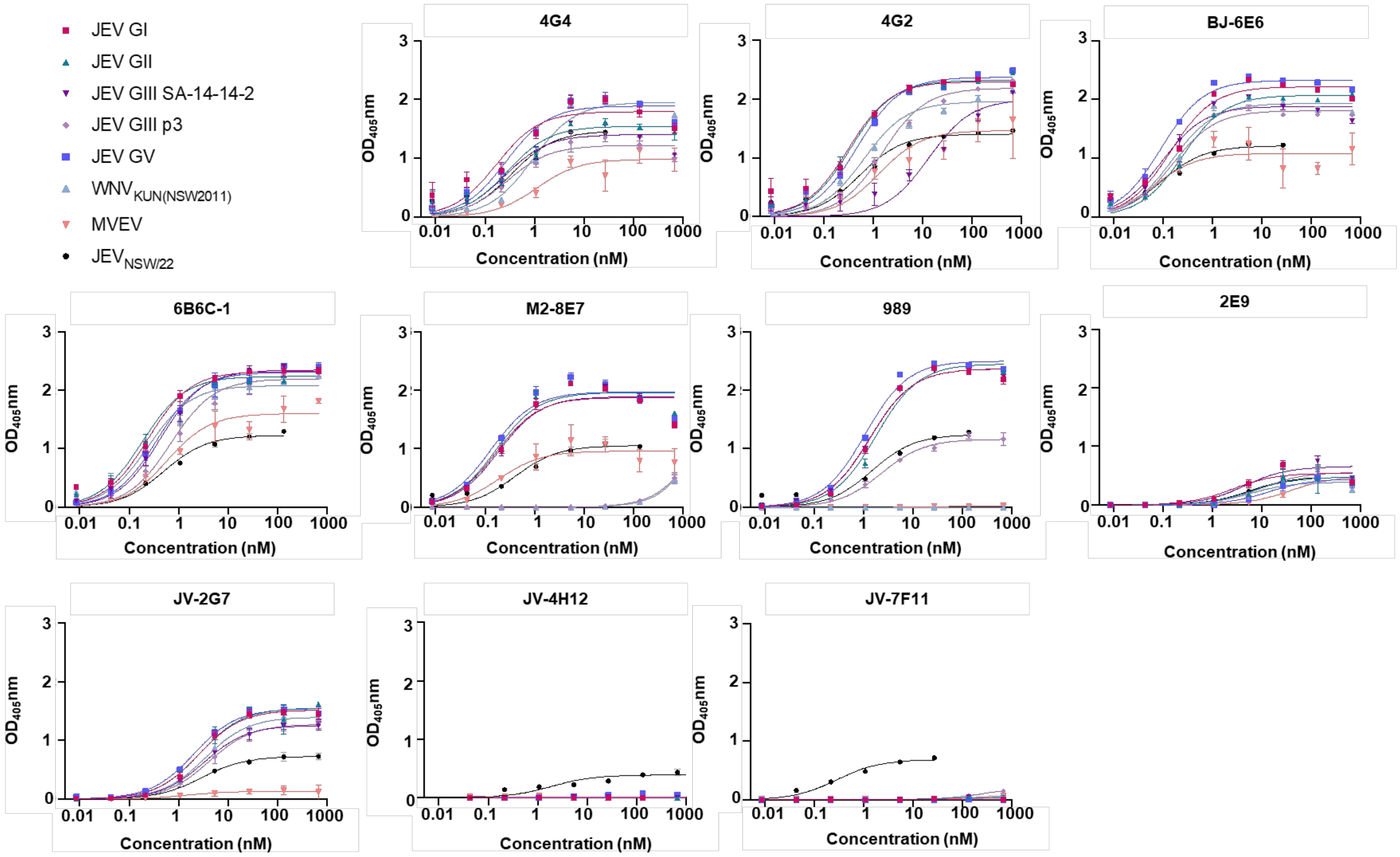

Supplementary Figure 3: K_d binding curves for mAbs to BinJ/JEV-prME genotypes I-V. Selected mAbs were titrated in a 5-fold dilution series starting from 10-100 µg/mL on C6/36 cell monolayers infected with BinJ/JEV-prME genotypes I-V, wild-type MVEV or $WNV_{KUN(NSW2011)}$ or mock-infected. The Kd binding curves of the data that was presented in Figs 2D and 2F.
